# Decarburization and Its Effects on the Properties of Plasma-Nitrided AISI 4140 Steel: A Review

**DOI:** 10.3390/ma18102207

**Published:** 2025-05-10

**Authors:** Željko Stojanović, Bojan Gligorijević, Slavica Prvulović, Adrian But, Petr Svoboda, Ján Piteľ, Aleksandar Vencl

**Affiliations:** 1 University of Novi Sad, Technical Faculty Mihajlo Pupin, Đure Đakovića bb, 23000 Zrenjanin, Serbia; stojanovic.zeljko@tfzr.rs (Ž.S.); slavica.prvulovic@tfzr.rs (S.P.); 2 University of Belgrade, Innovation Center of Faculty of Technology and Metallurgy, Karnegijeva 4, 11120 Belgrade, Serbia; 3 Faculty of Mechanical Engineering, Politehnica University of Timișoara, Bulevardul Mihai Viteazu 1, 300222 Timișoara, Romania; adi.but@gmail.com; 4 Faculty of Mechanical Engineering, Brno University of Technology, Technická 2896/2, 616 69 Brno, Czech Republic; petr.svoboda@vut.cz; 5 Faculty of Manufacturing Technologies, Technical University of Košice, Bayerova 1, 080 01 Prešov, Slovakia; jan.pitel@tuke.sk; 6 University of Belgrade, Faculty of Mechanical Engineering, Kraljice Marije 16, 11120 Belgrade, Serbia

**Keywords:** decarburization, plasma nitriding, low-alloy steel, friction, wear, corrosion

## Abstract

Despite the surge in plasma nitriding research, few reviews thoroughly examine how surface decarburization—occurring both before and during the treatment—affects AISI 4140 and similar steel grades. This review addresses that shortfall by providing an investigation into the decarburization process and its consequences. It compiles essential findings from prior studies, demonstrating instances of decarburization in both plasma-free and plasma-containing environments. Furthermore, this analysis explores strategies to prevent decarburization and assesses its significant impact on the steel’s microstructure, hardness, corrosion resistance, and wear properties in surface and near-surface regions. Moreover, this study proposes directions for future research, emphasizing the necessity for a more detailed understanding of the decarburization mechanisms and their influence on the properties of plasma-nitrided steels.

## 1. Introduction

AISI 4140 is a type of steel frequently subjected to plasma nitriding. This steel is commonly used in the automotive and chemical industries due to its high strength and low cost, as well as its high hardenability, impact toughness, and wear resistance [1,2,3]. Prior to plasma nitriding, this steel is typically subjected to heat treatment (quenching and tempering) and plasma cleaning. Table 1 shows the nominal chemical composition of AISI 4140 steel [2]. In general, the goal of quenching and tempering of AISI 4140 and similar steel grades is to produce a hard surface layer and a tough core in the steel parts, with a relatively uniform and stable surface structure suitable for nitriding. Plasma cleaning, on the other hand, is performed to remove contaminant species from the surface of the steel that typically form and accumulate during the previous stages of surface preparation. Surface contaminants, such as oil, grease, and/or traces of ink or paint, are removed to reduce the risk of arc formation and unstable plasma glow discharge during plasma nitriding [4,5].

It is well known that heat treatment, plasma cleaning, and plasma nitriding may all cause a phenomenon known as “surface decarburization” of steel [6,7,8,9,10,11]. According to the ASM handbook, surface decarburization of steel is defined as the depletion of carbon from the surface layer of a carbon-containing alloy due to its reaction with chemical substances present in the surrounding environment [12]. Although surface decarburization has been widely studied, recent research [13,14,15] on the effects of the oxide layer structure in 60Si2Mn steel [14] and the influence of alloying elements and water vapor content in transformation-induced plasticity (TRIP) steels [15] underscores the need for further investigation. These studies indicated that key factors influencing decarburization are not yet fully understood, highlighting the necessity for continued research to refine prevention strategies and enhance material performance. Previous research has shown that the loss of carbon significantly affects the surface microstructure of steel [16,17], its strength [18,19,20], ductility [21], hardness [22,23], corrosion resistance [24], wear resistance [25,26], and fatigue performance [27]. This effect is particularly significant in medium carbon steels, such as AISI 4140 steel, and becomes more prominent in steels with a higher carbon content [28,29].

The existing literature lacks comprehensive review articles addressing the effects of surface decarburization before and during plasma nitriding of AISI 4140 and similar steel grades. To fill this gap, the present article aims to provide an analysis of this phenomenon and its implications. It highlights key findings from previous research, offering examples of surface decarburization in both plasma-free and plasma-containing environments. Additionally, this review discusses effective prevention strategies and examines the critical influence of decarburization on the microstructural, hardness, corrosion, and tribological properties of surface and near-surface regions of the steels under consideration. Finally, this study outlines potential directions for future research, emphasizing the need for a deeper understanding of the decarburization mechanism and its impact on the plasma nitriding process.

## 2. Surface Decarburization in Plasma-Free Environments

### 2.1. Properties of Steel Surface and Near-Surface Regions

**Figure 1:**
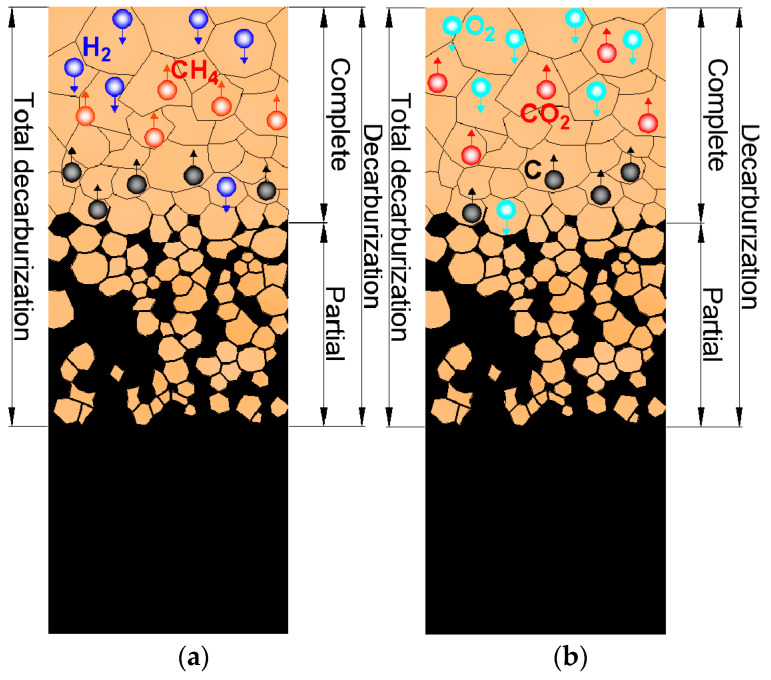
Transition from complete to partial decarburization caused by (**a**) H_2_ and (**b**) O_2_ in plasma-free environment. Decarburization begins at 790 °C in a 100% H_2_ atmosphere, at temperatures above 700 °C in a 2% O_2_ atmosphere, and at temperatures above 570 °C in the ambient air.

Mayer-Sidd and Hutterer demonstrated as early as in 1938 that decarburization of steel surfaces in plasma-free environments occurs due to prolonged exposure of steel to ambient air [30]. However, subsequent advancements in research revealed that surface decarburization of steel occurs during annealing when it is exposed to environments that contain oxygen (O_2_), carbon dioxide (CO_2_), hydrogen (H_2_), and/or water vapor (H_2_O) at elevated and high temperatures. Under these conditions, carbon on the steel surface reacts with gases, forming CO_2_, CH_4_, and/or CO [31,32,33,34,35]. Figure 1 illustrates the removal of C atoms from the steel surface driven by the action of H_2_ (Figure 1a) and O_2_ (Figure 1b). It also depicts the transition from complete (surface and near-surface) to partial (interior) decarburization in both cases (H_2_ and O_2_). In addition to atmospheric conditions [36,37,38,39,40,41,42,43,44], various factors influence the surface decarburization of steel, including alloying elements [17,45,46,47,48,49] and different temperatures and time intervals [50,51,52].

#### 2.1.1. Hydrogen-Containing Environments

Authors Fletcher and Elsea [53] referred to the study of Johansson and von Seth, noting that in 1881, Forquignon was the first to discover that decarburization of steel occurs in H_2_-containing atmospheres. The authors further stated that Campbell exposed carbon and alloy steels to H_2_ at a pressure of 1 atm for a duration of 4 to 12 d. According to their report, after 12 d at temperatures ranging from 950 to 1050 °C, the carbon content in the 4Cr-5Mo steel decreased from 1.67 to 0.22 wt.%. It was noted that this steel contained a significant amount of two carbide-forming elements that make more stable carbides than the iron element.

Exposure of AISI 4340 steel to low-pressure H_2_ environment at 400 °C, conducted by Krishnan et al., yielded negligible results, as the steel sample does not change its microstructure at this temperature [54]. At 600 °C, the sample softens with the presence of surface decarburization. The surface is composed of ferrite grains, whereas the amount of ferrite increases with longer exposure times. At 900 °C, decarburization by H_2_ occurs rapidly. After 1 h, the structure consists of a mixture of ferrite and martensite. After 2 h, it is primarily composed of ferrite. After 7 h, the carbon content decreases to 0.05 wt.%. Egert et al. [55] referred to the study of Manory [56], who discussed decarburization prior to plasma nitriding. It was noted that reactions involving H_2_ remove O_2_ and C, forming a ferritic surface.

#### 2.1.2. Oxygen-Containing Environments

**Figure 2:**
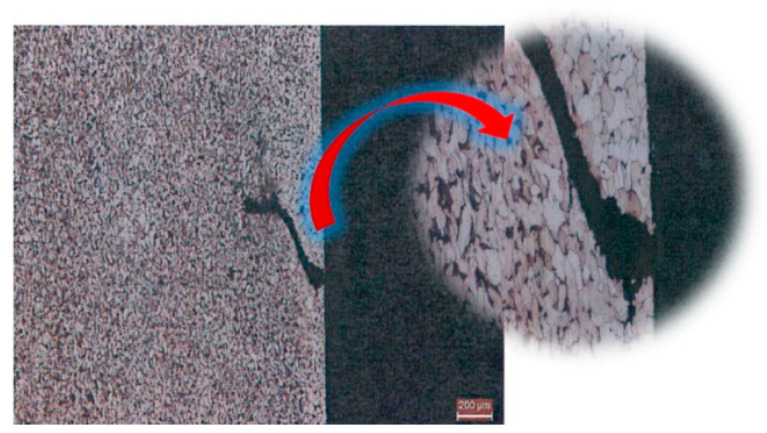
The surface crack and decarburization in the zone around the crack. Reproduced from [57] with permission from Springer Nature.

Although it does not provide precise details of the previous heat treatment conditions, research conducted by Deng et al. [57] revealed that the structure of ferrite and pearlite caused the fracture of the decarburized automotive casing made of SCM 420 steel (AISI 4118). The ferritic layer indicated that the carbon content at the outer surface is lower compared to the standard level. Likewise, a decarburized layer was present on the outer surface as well as around the crack, which is claimed to have contributed to crack formation (Figure 2).

**Figure 3:**
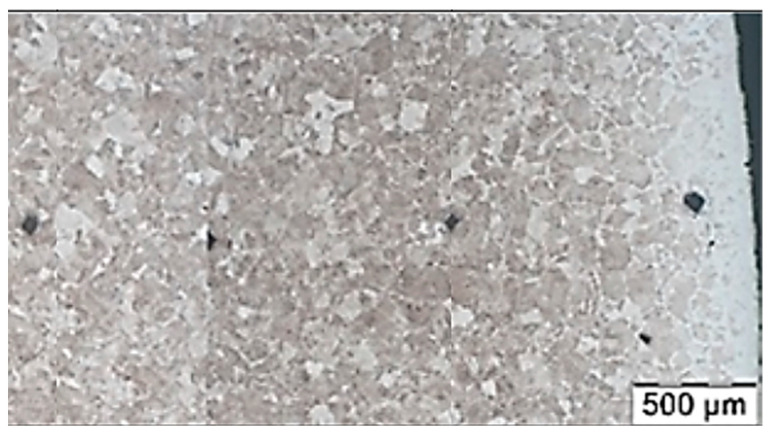
The microstructure of forgings for the annealing temperature of 620 °C and isothermal holding time 1 h. Adapted from Zwierzchowski [59], licensed under CC BY-NC 4.0 (https://creativecommons.org/licenses/by-nc/4.0/deed.en, accessed on 5 May 2025).

**Figure 4:**
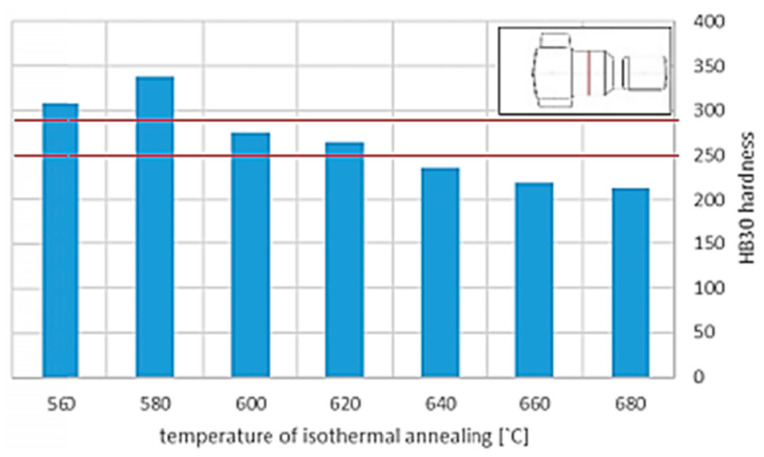
Medium Brinell hardness obtained for the analyzed forgings as a function of annealing temperature. Reprinted from Zwierzchowski [58], licensed under CC BY-NC 4.0 (https://creativecommons.org/licenses/by-nc/4.0/deed.en, accessed on 5 May 2025).

Similar changes were discovered by Zwierzchowski, who reported a slightly decarburized layer on AISI 4140 steel forgings of an adapter, the structure of which comprised pearlite and fine ferrite grains formed during the isothermal annealing process (Figure 3) [58]. The study found that the average hardness for isothermally annealed forgings at 600 °C is 275 HB, while for annealed forgings at 620 °C, it is 265 HB (Figure 4).

A slightly decarburized structure was also noted by García Navas et al. [59]. In their study on the effects of forging and heat treatment on the production of gears made of AISI 4140 steel, a higher presence of ferrite in the outer surface layers of the examined samples was confirmed. Forging at 1250 °C caused decarburization. The material consisted of pearlite/bainite and some retained austenite after forging. Notably, decarburized layers that formed during forging cannot be eliminated through subsequent heat treatment but by machining during later stages of the production process. However, if there is no subsequent machining, the decarburized layers remain, causing a reduction in tensile strength and product service life. After heating 42CrMo4 steel (AISI 4140) at 800–900 °C for 3–4 h, Calliari et al. [60] obtained a fully ferritic surface layer with coarse ferrite grains, while inner, partially decarburized layer exhibited a mixed ferrite–carbide structure.

**Figure 5:**
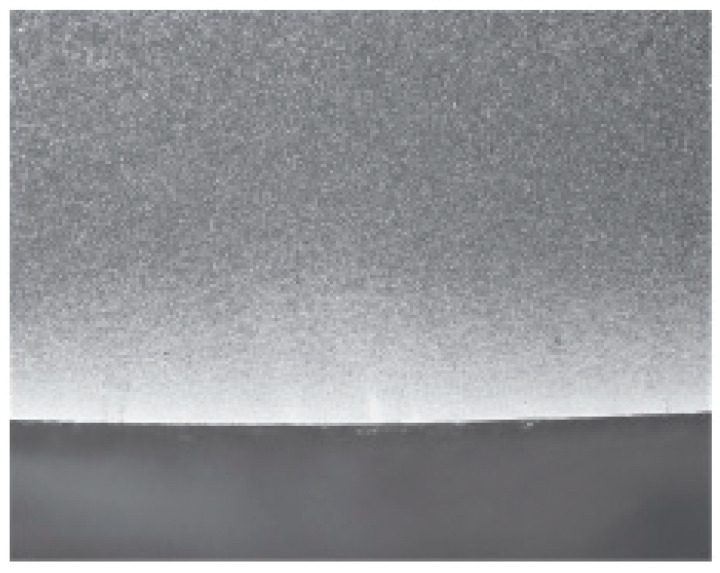
Micromorphology of carbon inclusions near the fracture surface depletion near the fracture origin. Reprinted from Hongfei et al. [61], licensed under CC BY 4.0 (https://creativecommons.org/licenses/by/4.0/, accessed on 5 May 2025).

**Figure 6:**
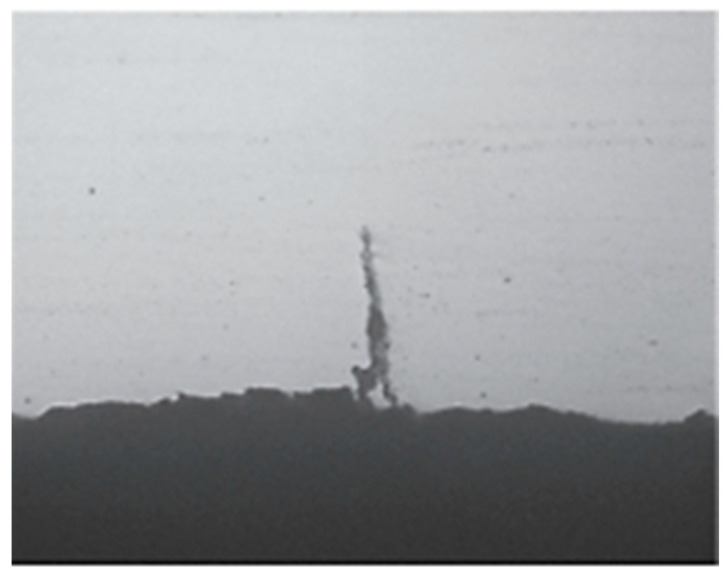
Magnified micromorphology of small distributed cracks in fracture origin area. Reprinted from Hongfei et al. [61], licensed under CC BY 4.0 (https://creativecommons.org/licenses/by/4.0/, accessed on 5 May 2025).

In a study on the fracture behavior of bolts made of 42CrMo steel, Hongfei et al. [61] noted a surprising reduction in the carbon content around the fracture site, which they attributed to the partial decarburization effect. Although the carbon-depleted layer was only 0.40 mm deep (Figure 5), they considered this layer of decarburization responsible for the bolt failure. Their claims assume that the presence of the decarburized layer reduces the dynamic strength of the bolt surface, leading to the formation of a fatigue fracture in the weaker section of the material. Delving deeper into the analysis, they revealed that cracks continued to grow (Figure 6), gradually reducing the cross-section of the bolt with higher stress. A failure occurred when the stress exceeded the fracture strength of the material.

Gabrić et al. [62] determined that the lowest carbon loss in the steel 42CrMo4 (AISI 4140) during annealing in a furnace without a protective atmosphere is recorded at approximately 830 °C. At lower and higher temperatures, the intensity of this process is more pronounced. They explained this phenomenon by the decarburization process occurring through interstitial carbon diffusion, which depends on the number of vacancies in the steel lattice, most of which are present at both lower and higher temperatures.

Decarburization also occurs when steel is heated to temperatures between 700 and 850 °C in a humid atmosphere (H_2_O), during which carbon in steel reacts with gases containing oxygen and hydrogen [63,64]. According to Jeong [65], SCM440 steel (almost identical to AISI 4140) exhibits a decarburized surface layer with a thickness of 125 μm when thermally treated at 770 °C and cooled in air to 720 °C.

### 2.2. Complete and Partial Surface Decarburization

**Figure 7:**
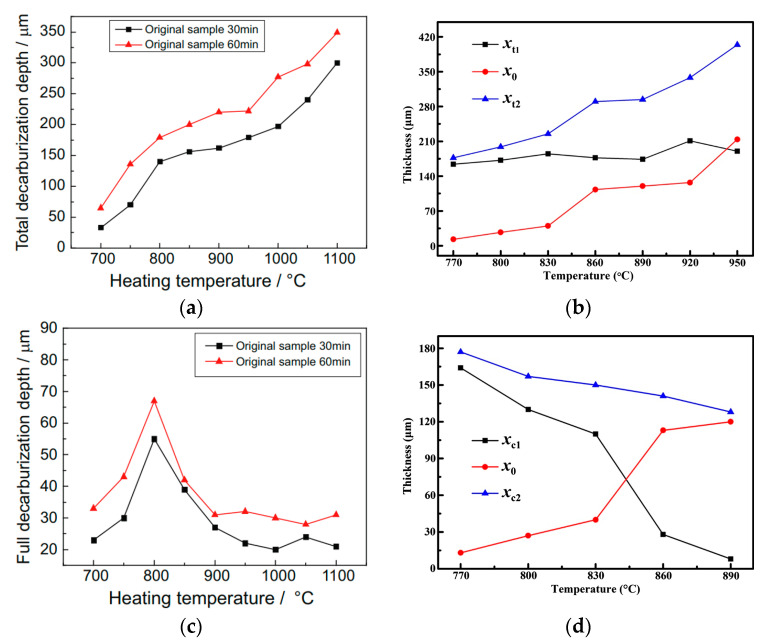
Variation in the depth of steel decarburization at different heating temperatures: (**a**) total decarburization, reprinted from Chen et al. [67], licensed under CC BY 4.0 (https://creativecommons.org/licenses/by/4.0/, accessed on 5 May 2025); (**b**) measured thickness of total decarburization layer (xt1), calculated thickness of oxidized iron (x0), and absolute thickness of total decarburization layer (xt2), reproduced from [68] with permission of Wiley; (**c**) depth of complete decarburization, reprinted from Chen et al. [67], licensed under CC BY 4.0 (https://creativecommons.org/licenses/by/4.0/, accessed on 5 May 2025); and (**d**) measured thickness of complete decarburization layer (xc1), calculated thickness of oxidized iron (x0), and absolute thickness of complete decarburization layer (xc2), reproduced from [68] with permission of Wiley.

During surface decarburization of steel, carbon atoms diffuse from the interior towards the surface of the steel. On their path, they react with hydrogen and/or oxygen species [66], producing carbon-containing gaseous products that transition from the steel into the surrounding gaseous environment. Chen et al. [67] and Duan et al. [68] investigated surface decarburization and oxidation of 35CrMo steel (AISI 4135). The authors demonstrated that the depth of the total (complete + partial) decarburization increases with increasing temperature (Figure 7a,b), with complete decarburization being dominant within the lower temperature range and partial decarburization being dominant within the higher temperature range (Figure 7c,d).

Totten et al. [69] stated that complete decarburization results in a fully ferritic microstructure in the surface layers, while partial decarburization is characterized by a gradual increase in carbon content from the ferrite layer to the core. This assertion is further clarified by the definition of decarburization proposed by Zhang et al. [70], based on the standards GB/T 224-2008 and ASTM E1077-01. According to these standards, complete decarburization is characterized by the presence of only ferrite, with grains adopting a columnar shape oriented perpendicular to the steel surface. In contrast, the structure of a partially decarburized layer consists of ferrite distributed along the boundaries of the prior austenite grains and pearlite. According to ISO 3887:2023 [71], complete decarburization, also called ferrite decarburization, refers to the distance from the product’s surface to the point where the carbon content stays below its solubility limit in ferrite, resulting in a purely ferritic structure. Partial decarburization refers to the distance from the point where the carbon content exceeds its solubility limit in ferrite—becoming distinguishable as structures like pearlite—to the point where it matches the core carbon concentration.

The formation mechanism of the completely decarburized layer on the surface of steel is influenced by phase transformations [67,68,72]. In general, at temperatures <A_c1_ (ferrite region, <745 °C), surface decarbonization is imperceptible. Only localized forms of decarburized surface layers are detected. In this temperature region, the surface decarburization rate is faster than the oxidation rate. The formation of a completely decarburized surface layer is limited by the dissolution of cementite, which is sluggish [72]. As a result, a smaller thickness of the completely decarburized surface layer is observed in this temperature range (Figure 7c). At temperatures >A_c3_ (austenite region, >810 °C), the oxidation rate tends to be equal or higher than the surface decarburization rate (Figure 7b,d). Surface decarburization is limited by the diffusion of carbon in austenite and oxygen in oxide layer. Completely decarburized surface layer forms as a phase transformation product from a carbon-depleted austenite during cooling. The narrow width of this austenite zone on the surface of the steel explains a shallower decarburization depth in this temperature range (Figure 7b). In addition, surface decarburization at lower temperatures within this austenite region (810–900 °C) produces both completely and partially decarburized surface layers (Figure 7c,d), whereas surface decarburization at higher temperatures (>900 °C) predominantly produces partially decarburized surface layer (Figure 7c,d). Within the A_c1_-A_c3_ region, known as the intercritical region (ferrite + austenite region), a completely decarburized surface layer is predominantly present. During surface decarbonization, the formation of this layer is controlled by the kinetics of the austenite-to-ferrite transformation, which is faster than the rates of limiting processes that control the formation of this layer outside this temperature range. This explains the peaking of the surface decarburization depth at 800 °C shown in Figure 7c. The presence of O_2_ in the surrounding gaseous environment causes the surface decarburization of AISI 4140 steel [73]. In addition, the presence of CO_2_ in the surrounding gas tends to form water vapor, which strongly promotes the surface decarburization. For this reason, CO_2_ partial pressure must be carefully controlled.

### 2.3. Prevention of the Surface Decarburization Effect

Controlling the atmosphere in the heating furnace is crucial for preventing surface decarburization of steel as the composition of the gaseous environment directly affects the intensity of this process. [74] Considering the errors that occur during hot processing of steel, authors Eugen Mayer-Sidd and Franz Hutterer noted that decarburization is particularly susceptible in high-carbon steels, as well as those containing tungsten and molybdenum [30]. The same authors indicated that the problem of surface decarburization can be avoided by packaging or using suitable protective gases during heating.

#### 2.3.1. Packaging

**Figure 8:**
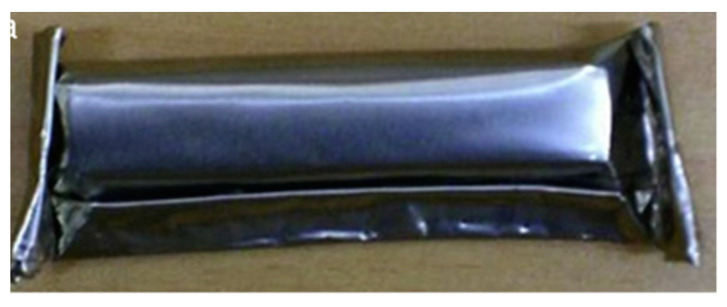
Protective packaging of parts: stainless-steel wrapping foil. Reprinted from Ramezani et al. [9], licensed under CC-BY-NC-ND 4.0 (https://creativecommons.org/licenses/by-nc-nd/4.0/, accessed on 5 May 2025).

**Figure 9:**
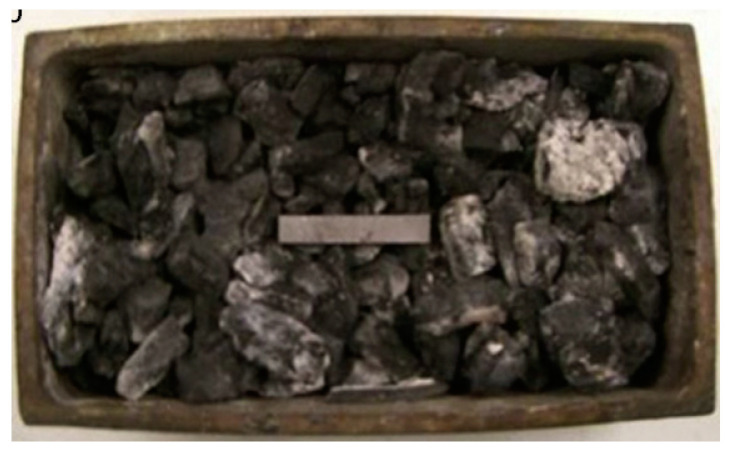
Protective packaging of parts: container for pack carburizing. Reprinted from Ramezani et al. [9], licensed under CC-BY-NC-ND 4.0 (https://creativecommons.org/licenses/by-nc-nd/4.0/, accessed on 5 May 2025).

Protective packaging of parts is an effective method for preventing the surface decarburization of steel by creating a physical barrier between the steel and the furnace environment through the wrapping of components in certain materials. This type of insulation reduces the need for the use of inert gas atmospheres in furnaces. As examples of prevention against the active surrounding atmosphere, stainless-steel wrapping foils and a sample packing container that is surrounded by charcoal are shown in Figure 8 and Figure 9, respectively.

**Figure 10:**
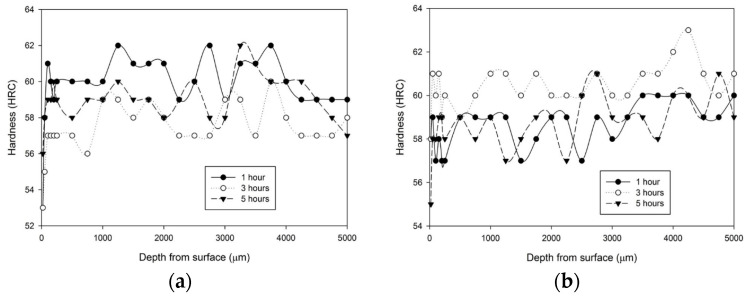
Hardness profiles of samples heat treated at 1020 °C with stainless-steel foil wrapping for 1, 3 and 5 h followed by: (**a**) fan cooling; (**b**) water quench. Reprinted from Pasang et al. [75], licensed under CC BY 4.0 (https://creativecommons.org/licenses/by/4.0/, accessed on 5 May 2025).

Pasang et al. showed that using stainless-steel foil to pack a sample of H13 steel can prevent the decarburization process from taking place in the vacuum of a muffle furnace, resulting in a constant hardness profile, ranging from 56 to 63 HRC. They demonstrated that the hardness varies between 1 and 2 HRC from the mean value, which is acceptable on the industrial scale [75] (Figure 10).

Prabhudev has claimed that active coal can be used to form an insulating cover that reduces heat loss and prevents surface decarburization. He has emphasized that processing parts in a muffle furnace without a neutral atmosphere are prone to this phenomenon [76]. Therefore, it is advisable to use a furnace with a controlled atmosphere. To prevent decarburization of AISI 4140 steel samples, Ambrosini [77] packed the samples in iron filings during austenitization at 845 °C for 50 min. During quenching, it is essential to adopt all possible measures to prevent surface decarburization and avoid a reduction in the dynamic strength and wear resistance.

#### 2.3.2. Protective Gases

Many authors have suggested the use of industrial gases that create a protective atmosphere in the furnace chamber and prevent the contact of steel with oxygen. The application of these gases achieves more complete protection against reactions with the active surrounding gaseous environment. When heat treatment is performed without the presence of an inert gas, it can lead to the formation of a decarburized layer. By adhering to this principle, Kwon et al. [78] avoided decarburization and achieved a fully martensitic structure in AISI 4340 steel, despite performing austenitization for 1 h in an argon atmosphere at temperatures of 870, 1030, and 1200 °C, respectively, followed by oil quenching. De Souza et al. [79] utilized a tubular furnace for quenching treatment with a controlled atmosphere of inert argon gas to avoid decarburization. Another example from the available literature is the study of Totten et al. [69]. They stated that an exothermic atmosphere (exogas) can be used as a protective atmosphere to prevent decarburization, provided that the dew point is minimized, to achieve the best results, which can be accomplished by drying the gas.

**Figure 11:**
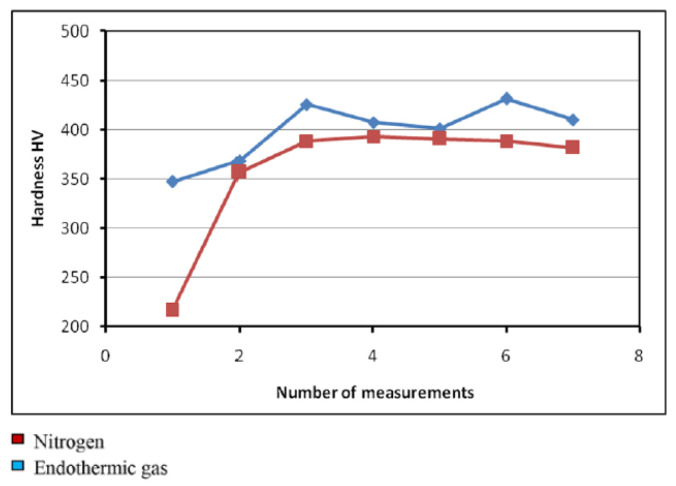
Hardness measurements performed after cross-sectioning of specimens heat-treated in nitrogen and endothermic gas. Reprinted from Prijanovič Tonkovič and Knez [80], licensed under CC BY-NC-ND 4.0 (https://creativecommons.org/licenses/by-nc-nd/4.0/, accessed on 5 May 2025).

**Figure 12:**
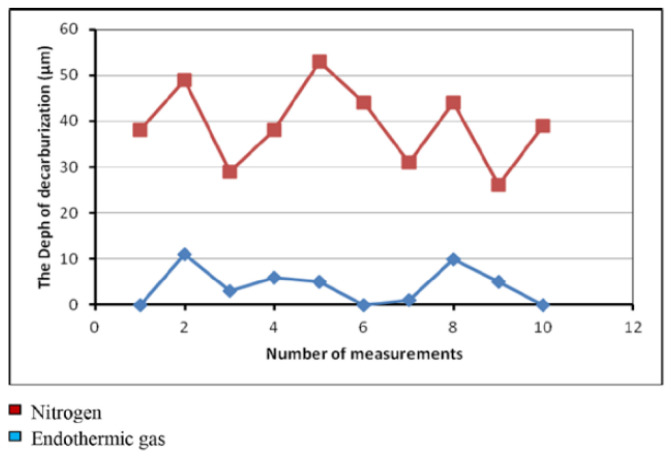
The depth of the decarburized layer. Reprinted from Prijanovič Tonkovič and Knez [80], licensed under CC BY-NC-ND 4.0 (https://creativecommons.org/licenses/by-nc-nd/4.0/, accessed on 5 May 2025).

Research analyzing the effects of protective atmospheres—endothermic gas and nitrogen—on the decarburization process of bolts made from 1.5510 steel has revealed that endothermic gas offers superior protection against decarburization compared to nitrogen [80]. The heating process was conducted at 830 and 870 °C, followed by quenching in mineral oil at 55 ± 5 °C and tempering at 510 °C for 60 min. Specimens treated in a nitrogen atmosphere showed lower hardness (36.67 HRC) compared to those treated in endothermic gas (38.44 HRC). Vickers hardness testing (HV 0.3) further confirmed this trend, with hardness values ranging from 347.2 to 431.8 HV for specimens treated in endothermic gas and hardness values ranging from 216.4 to 413.4 HV for those treated in nitrogen (Figure 11). Microstructure analysis revealed a maximum decarburized layer depth of 70 μm in specimens treated in a nitrogen atmosphere, with a ferritic microstructure on the surface (Figure 12). In contrast, specimens treated in endothermic gas exhibited a maximum decarburization depth of 10 μm, with the presence of a thin ferrite surface layer [80].

## 3. Surface Decarburization in Plasma-Containing Environments

### 3.1. Plasma-Cleaning and Plasma-Nitriding Environments

Plasma cleaning is typically applied to a steel surface before plasma nitriding to remove contaminants accumulated during previous surface preparation stages. This process relies on three main phenomena that lead to surface cleaning: heating, sputtering, and etching [81].

Heating is the most straightforward plasma-cleaning method [81]. A surface exposed to plasma absorbs heat primarily through electron and ion bombardment, as well as plasma radiation. Applying a positive or negative bias voltage to the substrate relative to the plasma potential increases electron and ion energy fluxes. Although heating is often impractical and costly in industrial settings, it remains an effective method for removing adsorbed moisture from surfaces. The process becomes more efficient when utilizing heat generated by plasma electrons. This method was used to dry large vacuum vessels and even the interiors of gas cylinders. Electron bombardment delivers energy directly to the surface, eliminating the need to heat the entire object to high temperatures.

Sputtering is one of the most widely used cleaning methods [81]. It occurs when external voltage is applied between the plasma and the surface being cleaned. A conductive object placed in plasma typically develops a “floating potential” of 10–20 V. However, the sputtering threshold for most metals, as well as their oxides and nitrides, is approximately 30 V or higher. The floating potential is typically insufficient to accelerate ions from the plasma and initiate sputtering. Sputtering occurs only when an additional voltage is applied between the plasma and the object. In this process, positive ions—mainly nitrogen or inert gases like argon—bombard the metal surface, removing atoms and contaminants to prepare it for nitriding. All atoms can undergo sputtering, but the sputtering yield depends on the surface composition and contaminant type. As a nonselective process, sputtering is not always an efficient cleaning method. Additionally, it removes some bulk material and introduces defects, especially in the final stages when only a few contaminant layers remain. Despite these drawbacks, plasma cleaning by sputtering is widely used in applications where plasma etching and heating alone cannot achieve the required level of cleanliness. Roliński et al. [82] considered sputtering a positive phenomenon, especially in the context of enhancing the surface activation process, noting that ejected atoms react with gas molecules and redeposit onto the cathode. During sputtering, in a plasma gas (most commonly ionized nitrogen species or a mixture of ionized nitrogen and hydrogen species), positively charged ions are accelerated toward the cathode (steel workpiece) under the influence of an electric field. When these ions make contact with the steel, they transfer their kinetic energy to the atoms present on its surface. If this kinetic energy is sufficient, the ions from plasma gas cause the ejection of the surface atoms into the surrounding atmosphere. In this regard, heavier ions from plasma gas, such as nitrogen ions, carry more kinetic energy and are more likely to cause a sputtering event compared to lighter ions.

In plasma cleaning by etching, reactive atoms or radicals from the plasma interact with the surface through chemical reactions [81]. Initially, these species adsorb onto the surface, influenced by chemical affinity and temperature. Some react with surface atoms to form new compounds, while others desorb before a reaction occurs. If the resulting compounds are volatile, they transition into the gas phase and are removed by the vacuum system. The desorption of volatile products requires activation energy; so, its rate increases with temperature. However, excessive heat can cause reactive plasma species to desorb before they interact with the surface. Typically, an optimal temperature maximizes the etching rate while allowing for some surface heating, which may be acceptable or even beneficial.

Before plasma nitriding, steels are often cleaned in a hydrogen-containing plasma, where the process functions as plasma etching. When only heavier gases like argon or nitrogen are present, sputtering dominates the cleaning process. If both hydrogen and heavier gases are used, cleaning occurs through sputter-etching. Since plasma cleaning of steel surfaces is typically conducted at elevated temperatures, the heating phenomenon may enhance the effectiveness of both sputtering and etching. During plasma nitriding of steels, nitrogen is usually mixed with hydrogen in various ratios, allowing both sputtering and etching to take place simultaneously.

#### 3.1.1. Surface Decarburization in Plasma-Cleaning Environments

Plasma cleaning is widely used to remove surface contamination from steel, enabling nitrogen species to interact with the surface during subsequent plasma nitriding. This ensures uniform nitriding across the entire steel surface. For steels, plasma cleaning is often performed in a hydrogen environment. Hydrogen’s presence can significantly influence surface decarburization, impacting the final properties of the steel. This effect has been largely overlooked in the literature, with only a few studies exploring the issue.

Sirin and Kaluc [83] and Kurny [84] referenced Edenhofer’s study [85], which demonstrated that during plasma nitriding, sputtering removes carbon along with other elements from the steel surface, thereby reducing the surface carbon content in a carbon-free plasma gas. In contrast, Miyamoto et al. [86] demonstrated that iron and contaminant species are removed from the steel surface through sputtering and/or etching (chemical reduction with hydrogen). Molecular hydrogen reacts with surface oxides, creating a clean surface before plasma nitriding. In a separate study, Tier et al. [87] reported that the low carbon potential in the surface region of AISI M2 steel results from carbon removal through sputtering and etching with H_2_. Similarly, Ruset et al. [88] demonstrated significant decarburization of C45 steel when the plasma atmosphere contains only H_2_. Kwietniewski et al. [89] made comparable observations. Before plasma nitriding, etching of AISI M2 high-speed steel was conducted at 150 °C for 60 min in a hydrogen atmosphere. This process resulted in significant decarburization, as observed in the carbon concentration depth profiles. Additionally, Rad et al. [90] conducted etching of AISI H11 steel in a hydrogen atmosphere for 15 min. They reported a reduction in the intensity of diffraction peaks associated with the ε phase, while the γ’ phase content increased. Another study that discussed the surface decarburization effect under these conditions is the study of Yao [91]. This author referred to the results of Krishnan [92], who found that a certain amount of surface decarburization can occur when AISI 4340 steel is exposed to hydrogen atoms at a temperature of 500 °C.

#### 3.1.2. Surface Decarburization in Plasma Nitriding Environments

Many studies reported that in plasma-containing environments, bombardment by positive ions at elevated temperatures leads to surface decarburization of the steel [93,94,95,96,97,98,99,100,101,102]. Although these ions play a crucial role in surface activation prior to plasma nitriding, as mentioned in the previous section, they also contribute to carbon depletion from the steel surface during plasma nitriding. Ruset et al. [88] confirmed that decarburization is directly influenced by sputtering during plasma nitriding [103,104,105,106,107,108,109,110,111,112]. They argued that processing parameters that promote high sputtering rates contribute to intense decarburization, leading to a significant reduction in surface carbon concentration on the workpiece (cathode). This results in the formation of a single-phase γ’-Fe_4_N layer, as predicted by the Fe-N-C phase diagram [113]. However, when plasma nitriding is conducted at elevated pressures (>5.2 mbar or 520 Pa) or with a high nitrogen content in the plasma atmosphere, the sputtering rate becomes insufficient to induce significant decarburization [88]. As a result, a considerable amount of the ε-Fe_2_-_3_(N,C) phase forms alongside γ’. In contrast, when the plasma-nitriding chamber contains only hydrogen (H_2_), decarburization is highly pronounced. A slightly reduced but still substantial decarburization effect occurs when 25% nitrogen (N_2_) is introduced into the plasma chamber, maintaining a sufficiently low surface carbon concentration, and promoting the formation of a monophase γ’ structure [114]. As the nitrogen content in the plasma gas increases to 80%, both sputtering and decarburization rates decline further. Plasma ions become ineffective at removing surface carbon, allowing carbon atoms to remain alongside nitrogen within the ε-Fe_2_-_3_(N,C) structure.

**Figure 13:**
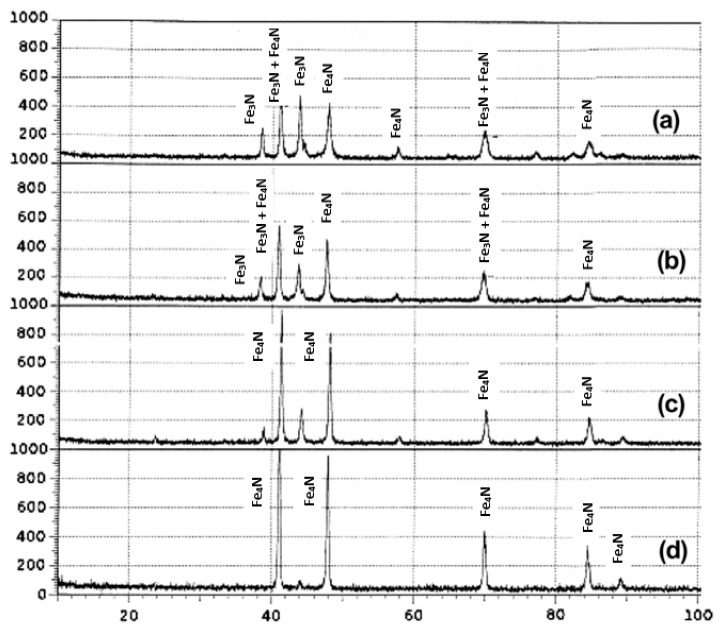
X-ray diffraction patterns of ion-nitrided AISI 4340 steel for (**a**) 500 °C/2 h; (**b**) 500 °C/16 h; (**c**) 540 °C/2 h; (**d**) 540 °C/16 h. Reprinted from [83], with permission from Elsevier.

Mohammadzadeh et al. [105] conducted plasma nitriding on steel samples treated under a floating potential. Their analysis revealed that the energy of ions reaching the steel surface is only around 15 eV. Since this energy is insufficient to induce sputtering and significant decarburization, the high surface carbon content favors the formation of ε nitride rather than γ’. Sirin and Kaluc [83] reported that at 500 °C, both ε-Fe_2_-_3_N and γ’-Fe_4_N phases are present. However, at 540 °C, the ε-Fe_2_-_3_N phase is no longer detectable. At this temperature, the compound layer consists exclusively of the γ’-Fe_4_N phase (Figure 13). The authors concluded that structural variations in the compound layer result from the combined effects of decarburization, denitriding, and sputtering.

After performing pulsed plasma nitriding of AISI H13 samples with a gas mixture of N_2_ and H_2_, Karimzadeh [93] reported that sputtering leads to surface decarburization of the cathode and confirmed that higher sputtering rates effectively facilitate the formation of the γ’ phase.

### 3.2. Effects of Temperature, Time, and Nitriding Potential on Surface Decarburization Phenomenon During Plasma Nitriding

**Figure 14:**
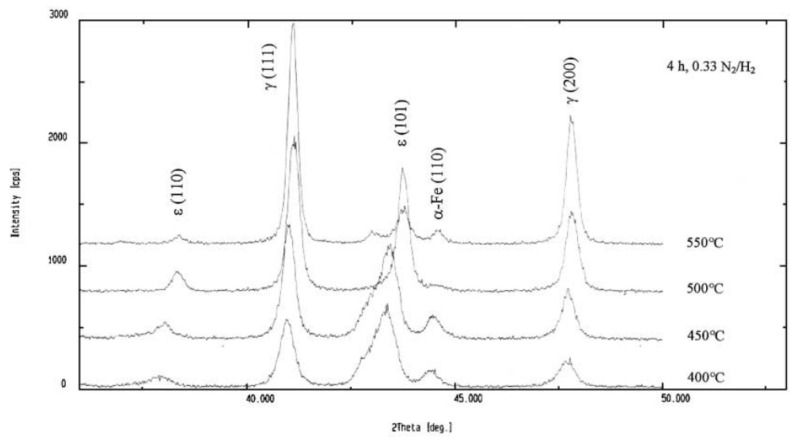
XRD patterns of AISI 5140 low-alloy steel for 4 h treatment time, for 0.33 N_2_/H_2_ gas mixture ratio, and at 400, 450, 500 and 550 °C temperatures. Reprinted from [103], with permission from Elsevier.

**Figure 15:**
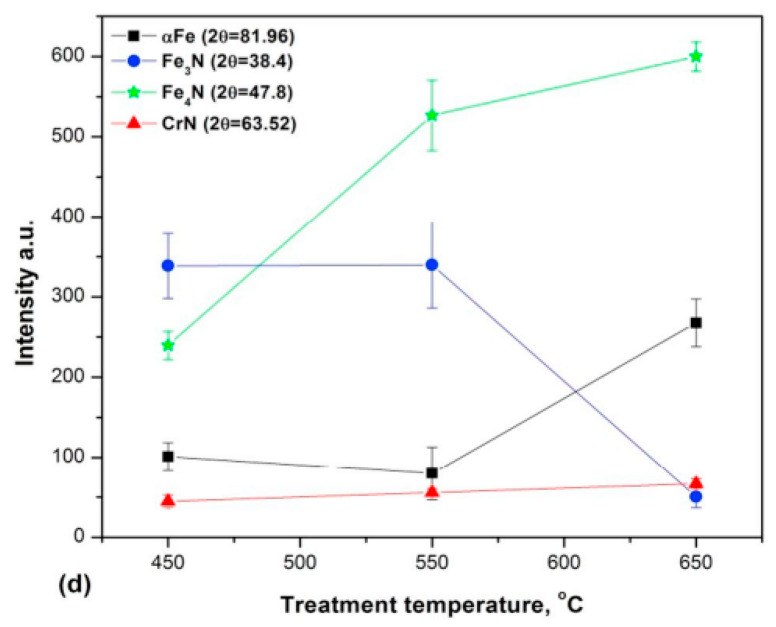
XRD patterns of AISI 5140 low-alloy steel for 4 h treatment time, for 0.33 N_2_/H_2_. Reprinted from [118], with permission from Elsevier.

Increasing the temperature and nitriding time while reducing the nitriding potential intensifies the surface decarburization effect. Wen [115] confirmed these findings, stating that the amount of ε nitrides in the compound layer (ε and γ’) decreases in samples nitrided at 550 °C compared to those treated at 400─500 °C due to sputtering during plasma nitriding. The author emphasized that when the nitriding temperature exceeds 525 °C or the duration exceeds 10 h, the ε nitride content declines due to decarburization and denitriding, as the sputtering rate surpasses nitrogen diffusion under these conditions. While some authors [110] support this explanation, attributing the reduction in ε phase content at higher temperatures to the rapid sputtering and decarburization that lower the surface carbon concentration, other authors have argued that this phenomenon is due to nitrogen’s lower diffusivity at lower temperatures. They have suggested that limited nitrogen diffusion at lower temperatures promotes the formation of a compound layer dominated by the ε-Fe_2-3_N phase, which explains the reduced presence of γ’-Fe_4_N at these temperatures [116,117]. Additionally, some researchers [118,119] have proposed that at higher temperatures, nitrogen diffusion into the material’s interior outpaces nitrogen adsorption on the surface, leading to the observed phase distribution. Figure 14 presents the XRD results of nitrided AISI 5140 low-alloy steel as a function of ion nitriding temperature, while Figure 15 illustrates variations in peak absolute intensity for selected phases. The results showed that higher treatment temperatures reduce the ε nitride content while increasing the γ’ nitride presence.

**Figure 16:**
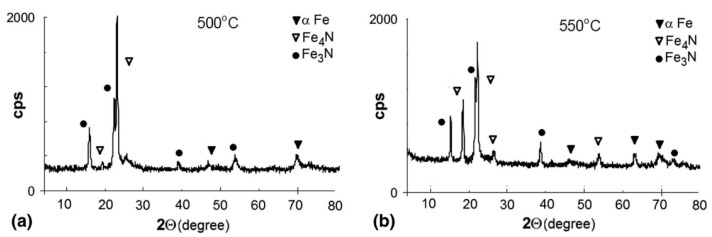
XRD patterns of the samples nitrided at (**a**) 500 and (**b**) 550 °C for 5 h. Reproduced from [121] with permission from Springer Nature.

Roliński and Sharp [120] conducted plasma nitriding on 39CrMo steel. They observed that the intensity of diffraction peaks that come from the ε phase decreases rapidly after 25 h of nitriding. After 400 h, the steel surface consists entirely of a single-phase γ’ compound layer. The authors attributed this behavior to carbon diffusion toward the surface during shorter nitriding durations, where carbon reacts with iron and nitrogen to form the ε phase. However, during prolonged nitriding, sputtering removes carbon from the surface, allowing nitrogen to replace it, leading to sustained decarburization. Zdravecká et al. [121] reported similar findings in their study on 31CrMoV9 steel. They found that at higher temperatures and longer nitriding durations, a thinner white layer formed due to sputtering and decarburization. At 570 °C, these effects are responsible for the reduced thickness of the compound layer. Their analysis of samples nitrided at 500 °C showed a surface containing predominantly the ε-Fe_2-3_N phase, with a smaller fraction of γ’-Fe_4_N. The results further indicated that the γ’-Fe_4_N was present throughout the compound layer at 550 °C (Figure 16), while a monophase α-Fe(N) structure was found beneath the compound layer.

Mahboubi et al. [122] conducted plasma nitriding on quenched and tempered En19 steel (equivalent to AISI 4140), resulting in the formation of two sublayers within the compound layer. The upper sublayer exhibited a uniform grain structure with cracks and pores, while the inner sublayer, adjacent to the diffusion zone, displayed a columnar grain structure. Their XRD analysis aligned with previous studies, showing that plasma nitriding at 450 °C leads to the formation of both ε and γ’ in the compound layer. However, at 550 °C, the γ’ becomes predominant, with only trace amounts of the ε phase. At higher nitriding temperatures, carbide coarsening and ferrite grain growth contribute to surface softening of the substrate.

### 3.3. Carbon-Rich Zone at the Nitriding Front During Plasma Nitriding

Sun and Bell [123] investigated the causes of carbon redistribution in the plasma-nitrided layer, attributing it to pressure stresses and nitrogen absorption. They described this mechanism as the diffusion of carbon atoms originally present in the steel toward stress-free areas, specifically the surface and nitriding front. This process leads to nitrided layer decarburization, the formation of a carbon-rich zone at the nitriding front, and the development of a grain boundary parallel to the surface [124]. More importantly, they linked carbon adsorption and redistribution to phase formation, stating that a monophasic γ’ nitride layer forms only at very high nitriding temperatures or under low nitriding potential. Conversely, decreasing the temperature and duration or increasing the nitriding potential promotes the formation of the ε nitride.

## 4. Influence of Plasma-Nitriding Parameters on Surface Hardness, Corrosion Performance, and Tribological Behavior of AISI 4140 and Similar Steel Grades

In mechanical engineering, plasma nitriding is mostly applied for the improvement of the corrosion and wear properties of materials, as well as their resistance to dynamic loading [125,126]. The plasma-nitriding process parameters, such as temperature, plasma gas composition, pressure, and treatment duration, significantly influence these properties. Suboptimal adjustment of parameters leads to uneven partitioning of nitrides in the nitrided layers, causing components to become more susceptible to deterioration of mentioned properties. Increasing the temperature of plasma nitriding results in a decrease in hardness due to a reduction in the content of *ε* nitrides and an increase in the content of γ’ nitrides. Such a turn of events facilitates the increase in both wear and corrosion rates, as the presence of a higher amount of *ε* nitrides provides more stable wear and corrosion resistance compared to γ’ nitrides.

**Figure 17:**
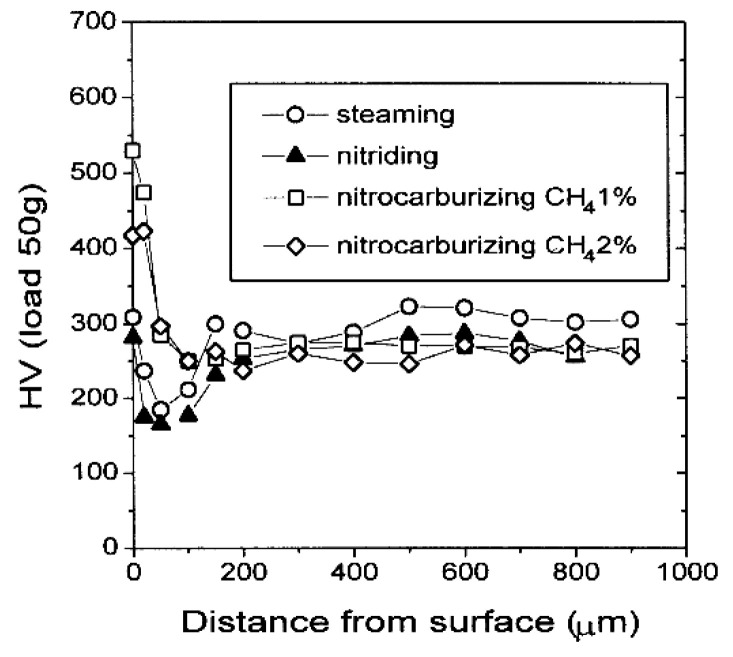
Hardness distributions of surface-treated sintered steels. Reprinted from [127], with permission from Elsevier.

**Figure 18:**
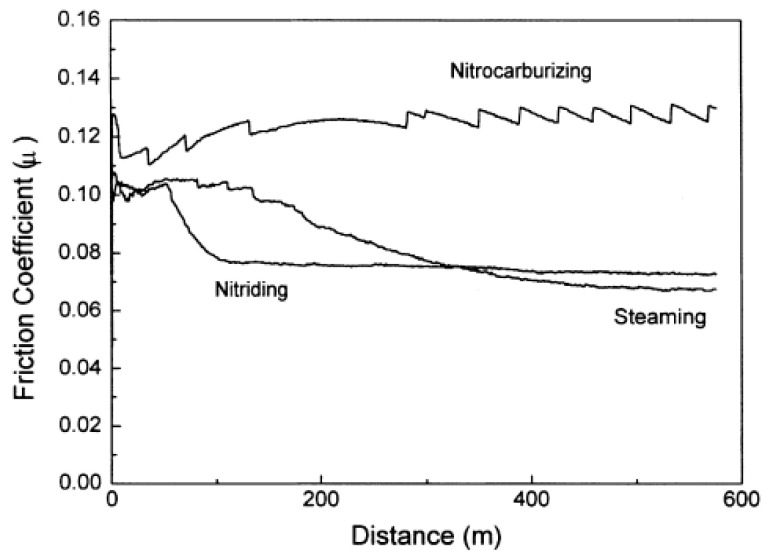
Variation in friction coefficients under tribological test with conditions of 875 N load and 0.25 m s−1 speed. Reprinted from [127], with permission from Elsevier.

**Figure 19:**
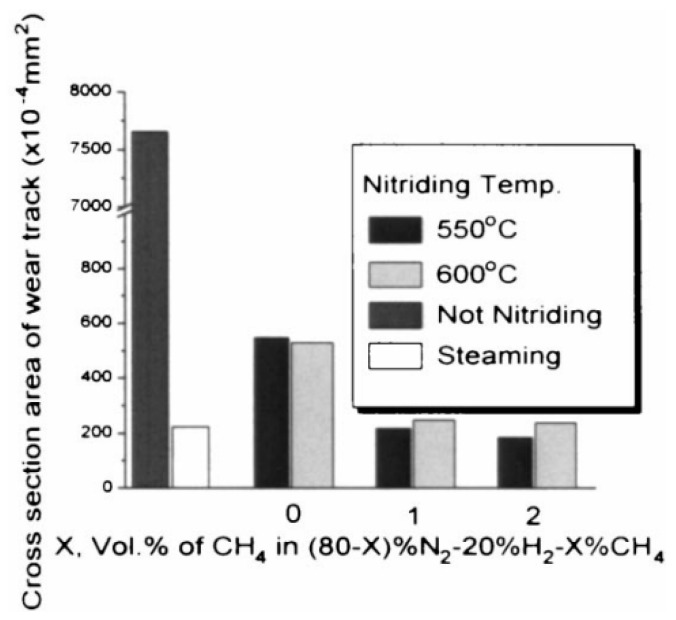
Wear test results for differently nitrocarburized specimens under controlled conditions: 875 N load and 0.25 m·s^−1^ speed. The value X = 0 corresponds to nitriding (N_2_:H_2_ = 80:20 vol%), while X = 1 and X = 2 represent nitrocarburizing with the addition of 1% or 2% CH_4_ to the gas mixture. Reprinted from [127], with permission from Elsevier.

Findings on the hardness behavior of sintered, plasma-nitrided steels were presented by Park et al. [127]. They applied plasma nitriding and nitrocarburizing processes at temperatures ranging from 500 to 600 °C. In their study, a ferrite phase up to 0.1 mm below the surface of the sintered steel was detected, which was ascribed to the surface decarburization phenomenon. Subsequent microhardness measurements demonstrated a decrease in hardness to a depth of 0.1 mm in the plasma-nitrided samples, which corresponded well with the previously observed microstructure (Figure 17). Variations in the coefficients of friction during tribological testing demonstrated that the nitrocarburized sample exhibits a higher coefficient of friction than the nitrided sample (Figure 18). The authors suggested that the results are primarily attributed to the reduction in the contact area between the pin and disk surfaces due to increased surface hardness, but this explanation is questionable. At low loads, the decrease in contact area reduces the adhesive component of friction and has a minimal effect on the deformation component. However, at high loads, the deformation component of friction, which increases as the contact area decreases, becomes dominant. The test conditions were relatively extreme, with a high load of 875 N and a low sliding speed of 0.25 m·s^−1^. The coefficient of friction values confirmed this, as they fall within the boundary lubrication regime (0.05–0.15) [128]. The value for the nitrocarburized sample was around 0.13, which is very close to dry-sliding conditions. Although the authors did not measure the counter-body wear, it may be expected that abrasive wear was also the dominant wear mechanism, suggesting that the deformation component of friction influenced the higher coefficient of friction observed. By testing abrasive wear using the pin-on-disc method under controlled conditions (Figure 19), the authors found that the wear of the nitrocarburized sample is less than that of the nitrided sample [127]. The reason for the improved wear resistance was attributed to the increased resistance to plastic deformation due to the enhanced hardness.

In a recent study, Landgraf et al. [129] agree with the findings of previous studies mentioned in this work that the composition of the compound layer is influenced by process parameters, i.e., that ε nitrides (Fe_2-3_N) occur at lower temperatures, while γ’ nitrides (Fe_4_N) appear at higher temperatures. In this regard, they further conclude that the reduction in surface hardness of the material at elevated temperatures is caused by a decrease in the content of ε nitrides. To obtain a monophase structure of γ’-Fe_4_N, Cesconetto et al. [130] nitrided API 5L X-70 steel in plasma gas for 5 h. They concluded that the formation of the γ’ structure occurs when the nitriding time is increased. The surface hardness of the nitrided layers, initially measuring 650 HV at 470 °C, begins to decrease to approximately 570 HV. The authors assigned this softening to decarburization or the transition of ε to γ’. In other words, high surface hardness can be achieved if a surface layer is predominantly composed of ε nitrides. By utilizing a temperature of 440 °C and a shorter nitriding time of 1 h, the authors achieved a compound layer predominantly composed of the ε-Fe_2-3_N, which exhibited the lowest wear factor (specific wear rate) of 1.30 *×* 10^−3^ mm^3^/Nm, thereby indicating the optimal conditions for achieving the highest wear resistance (Table 2). Prolonging the nitriding duration resulted in decreased wear resistance, attributed to microstructural changes and the formation of the γ’-Fe_4_N.

**Figure 20:**
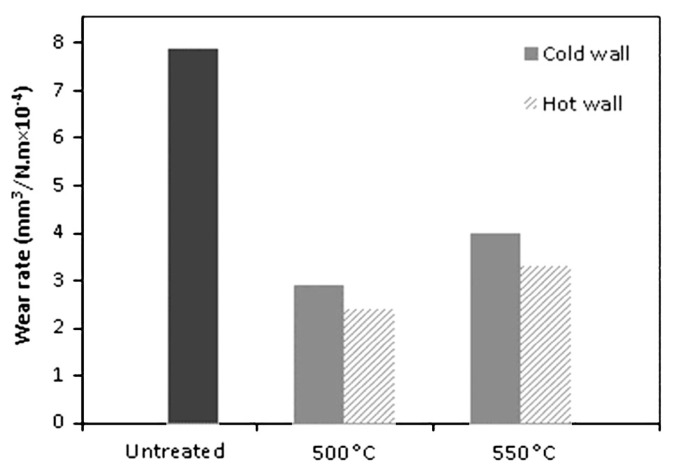
Wear rate of untreated and plasma-nitrided specimens. Reprinted from [101], with permission from Elsevier.

**Figure 21:**
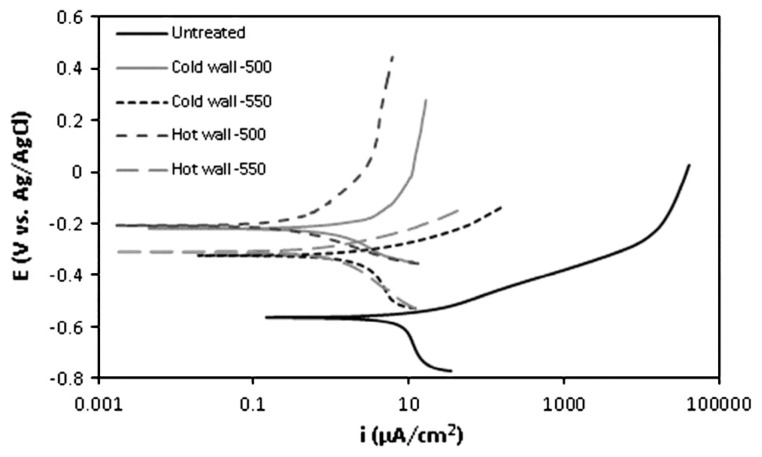
Potentiodynamic polarization curves for the untreated and nitrided specimens in 3.5 wt.% NaCl solution. Reprinted from [101], with permission from Elsevier.

The ε phase possesses a hexagonal close-packed (HCP) crystal structure, which, as noted by Maniee et al. [101], is harder than the face-centered cubic (FCC) γ’ crystal structure. The results indicated that the coefficient of friction increases with temperature increase due to the reduction in the ε phase content. The decrease in the coefficient of friction is influenced by the densely packed HCP structure of the ε phase, which causes poor adhesion of the steel sample and the counter-body surface. By increasing the nitriding temperature from 500 to 550 °C, the authors noticed an increase in wear rate, which was attributed to the reduction in the ε phase content in the compound layer of the sample nitrided at 550 °C (Figure 20). Decarburization can reduce the presence of the ε phase on the surface as the lack of carbon hinders the formation of this nitride. Conversely, a higher presence of the γ’ phase deteriorates the mechanical properties, considering that γ’ has a lower hardness and a higher tendency to wear compared to ε phase. An increase in nitriding temperature from 500 to 550 °C contributed to a decrease in corrosion potential, while the corrosion current density increases, leading to a decline in corrosion resistance. The reason for such corrosion behavior was assigned to the lower amount of ε nitrides in the compound layer of samples nitrided at 550 °C compared to those nitrided at 500 °C (Figure 21).

**Figure 22:**
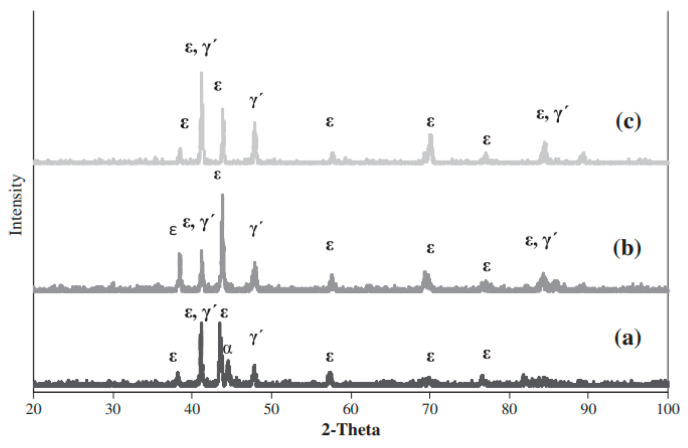
X-ray diffraction patterns of nitrided samples at (**a**) 530 °C, (**b**) 570 °C, and (**c**) 630 °C. Reprinted from [132], with permission from Elsevier.

Ebrahimi et al. conducted an investigation of the corrosion behavior of plasma-nitrided AISI 4140 steel. Their study included nitriding temperatures of 530, 570, and 630 °C, a process duration of 5 h, an atmosphere containing 80% N_2_ and 20% H_2_, and the application of sputtering using H_2_ [131]. Among all cases, the samples nitrided at 570 °C exhibited the highest corrosion resistance. These samples contained the largest amount of ε phase in the compound layer (Figure 22). XRD patterns revealed that the amount of ε phase increases as the nitriding temperature rises from 530 to 570 °C, but then decreases at 630 °C. The authors described the composition of the compound layer derived at 570 °C as the ε phase with traces of γ’ phase, while the compound layer obtained at 630 °C predominantly contained the γ’ phase.

The credibility of the research conducted by Ebrahimi et al. [131] is supported by Oliveira et al. [132]. They discussed that the ε phase exhibits superior corrosion resistance than the γ’ phase due to its crystal structure and higher nitrogen content. In addition, they concluded that the corrosion resistance of the nitrided layer depends on the composition of the compound layer.

Çelik and Karadeniz [133] recorded a significant decrease in the surface hardness of AISI 4140 steel as the plasma-nitriding temperature increased. The highest surface hardness was obtained during nitriding at 500 °C for 10 h. However, at higher temperatures (550–600 °C), the surface hardness decreased. They observed that the hardness at 600 °C (460–510 HV) significantly decreases compared to that at 500 °C (900–980 HV). Since the XRD analysis confirmed the presence of γ’ (Fe_4_N) at all plasma-nitriding temperatures, the authors suggested that this process should not be carried out at higher temperatures as it results in a reduction in surface hardness.

Plasma nitriding of Fe-Mo-C sintered steels at a temperature of 550 °C for more than 8 h produced a surface hardness of around 700 HV. A sharp drop in hardness to 450 HV was noticed beneath the compound layer after nitriding for 24 h [134]. XRD analysis demonstrated the predominant presence of γ’ nitrides in the samples nitrided at 550 °C, as well as the presence of ε nitrides to a lesser extent. Based on these findings, Molinari et al. [134] confirmed decarburization in samples exhibiting the absence of precipitation in the subsurface zones during plasma nitriding at 550 °C for 24 h. Agreement with previous findings is also found in the study of Zhang et al. [135]. Namely, these authors demonstrated that the wear resistance of the oxynitrocarburized layer decreases with the reduction in the ε-Fe_3_N phase. Table 3 presents the detailed conditions of this study along with its key findings.

**Figure 23:**
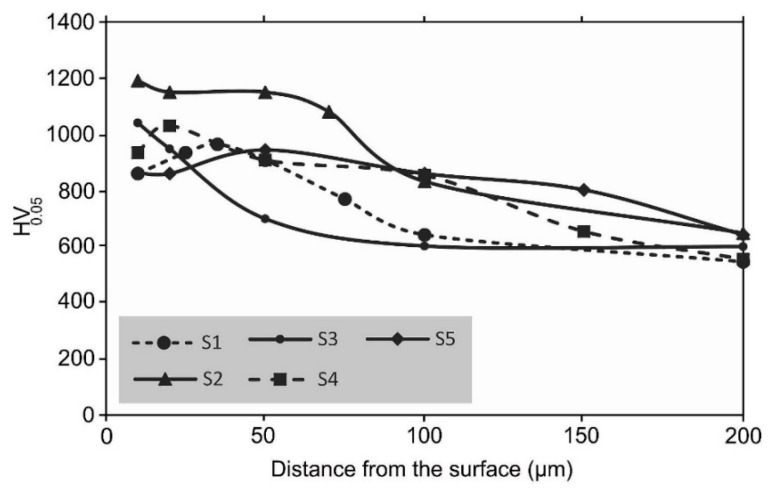
Hardness depth profile for plasma-nitrided specimens under different conditions: S1 (520 °C; 6 h), S2 (520 °C; 16 h), S3 (520 °C; 8 h), S4 (530 °C, 8 h), and S5 (540 °C, 8 h). Adapted from Karimzadeh et al. [93], licensed under CC BY-NC 4.0 (https://creativecommons.org/licenses/by-nc/4.0/deed.en, accessed on 5 May 2025).

**Figure 24:**
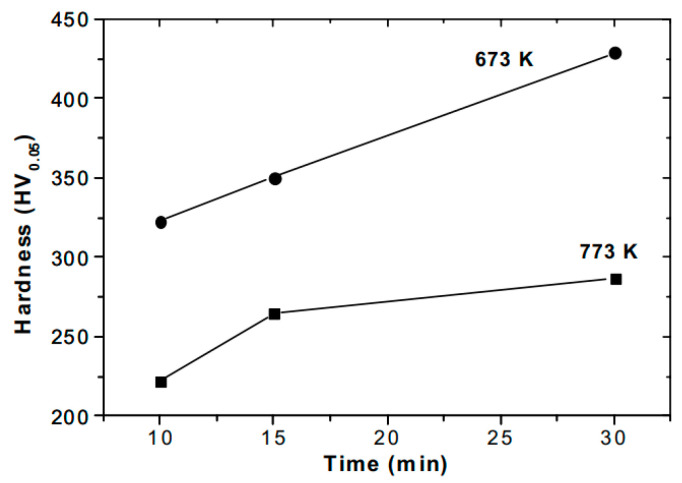
Surface hardness as a function of nitriding time for samples treated at 400 °C and 500 °C in H_2_-50%N_2_. Reprinted from Abdalla et al. [136], licensed under CC-BY-NC-ND 3.0 (https://creativecommons.org/licenses/by-nc-nd/3.0/, accessed on 5 May 2025).

The analyses of the influence of temperature on the mechanical properties of nitrides in hot-worked H13 steel, presented in [93], showed that an increase in the processing temperature contributes to a reduction in the near-surface hardness (sample S5 (540 °C, 8 h), Figure 23), which increases again with the passage of time and the depth of the layer (Figure 23). The authors attributed this behavior to the reduction in the amount of the ε phase and the increase in the amount of the γ’ phase. Like the above-mentioned studies, the authors showed that the amount of the ε phase influences the coefficient of friction because of its structure and high hardness. Variability in hardness was also recorded in Ref. [136], where Abdalla et al. observed a decrease in hardness in the case of AISI 1010 samples plasma nitrided at 500 °C relative to those nitrided at 400 °C. They attributed this drop in hardness to the significant decarburization that occurred during the pre-heating and cleaning, i.e., sputtering in a discharge containing H_2_-50%Ar for 30 min. (Figure 24).

## 5. Future Directions

The uncertainties regarding the influence of the surface decarburization on the properties of plasma-nitrided AISI 4140 steels indicate an incomplete understanding of this phenomenon. The lack of detailed investigations of surface decarburization of AISI 4140 steel indicates the need for a comprehensive approach in research on this subject to understand the interactions between plasma and steels being nitrided. Previous studies only mentioned/discussed surface decarburization of AISI 4140 or similar steel grades but often did not delve into its detailed examination [137,138,139,140]. In cases of other steel grades, the authors unanimously concluded that the mentioned atmospheres lead to the decarburization of the material’s surface, but they did not delve into the investigation of this phenomenon [40,141,142,143]. Here, it is important to emphasize that this issue has not been reported in previous research on plasma nitriding of AISI 4140 steel. Not only is there a noticeable lack of documented effects of surface decarburization on the properties of AISI 4140 steel, but there is also uncertainty regarding the potential effects of this process on the tribological and corrosion properties of these materials. The same can be concluded for similar steel grades.

Most studies investigated the process side of plasma nitriding by modifying parameters such as gas composition, duration, and temperature, while significantly fewer examined the role of material properties. There is a notable absence of research on the influence of surface and near-surface crystallography on the decarburization and plasma-nitriding behavior of AISI 4140 and similar steel grades. More generally, the impact of crystallographic characteristics on the hardness, corrosion resistance, and tribological performance of these plasma-nitrided steels remains insufficiently studied. Furthermore, the effects of other microstructural features are also poorly understood. These include the initial presence, morphology, and thermal stability of ε-carbide and/or cementite particles, as well as the morphology and composition of various matrix constituents such as martensite, bainite, and retained austenite. Addressing these aspects in future research is expected to provide valuable insights into the plasma-nitriding process of AISI 4140 and comparable steels.

To evaluate the effects of the plasma-nitriding process, the properties of plasma-nitrided steels have often been compared to the properties of these steels prior to plasma nitriding. However, in some cases, this state does not adequately reflect the true reference state for comparison. This is because the steels in this condition have not undergone the surface decarburization process, which is usually the case during preparation of steel surfaces for plasma nitriding. For example, the removal of steel surface contaminants in hydrogen-containing environments prior to plasma nitriding may introduce the surface decarbonization phenomenon. This alone will change the initial quenched and tempered state of the steel surface, rendering it inadequate to serve as a reference state for comparison. Therefore, besides the quenched-and-tempered state, future studies should consider this state as well when evaluating the effects of plasma nitriding on the properties of steels. This will undoubtedly improve the overall understanding of plasma nitriding of steels.

The present review study has clearly shown the positive effect of ε nitride on the hardness, tribological, and corrosion properties of plasma-nitrided steels in question. Besides the effects of plasma-nitriding parameters, such as plasma gas composition, temperature, and time, there is an absence of other innovative approaches for increasing the presence and stability of this phase in the near-surface regions of plasma-nitrided steels. This aspect can be significant for future investigations related to improving critical properties of plasma-nitrided steels.

Considering that surface decarburization may occur during quenching and tempering, plasma cleaning, and plasma nitriding, there are no studies that have quantified and compared the levels of surface decarburization achieved during these processes and to what extent they individually affect the final properties of plasma-nitrided steels. Moreover, there are no methods that have been proposed to differentiate between the effects of surface decarburization occurring during plasma nitriding and that occurring prior to plasma nitriding. Therefore, quantifying, differentiating, and measuring the effects of surface decarburization achieved during different stages of the plasma-nitriding process also remain challenging for the future investigations.

## 6. Summary and Conclusions

In the Introduction section, the present review study briefly introduces the definition of the surface decarburization process that can occur both before and during plasma nitriding of AISI 4140 and similar steel grades. It also shows the most fundamental examples where this phenomenon was observed in plasma-free and plasma-containing environments. In the next two sections, this study analyzes previous research studies dealing with surface decarburization in plasma-free and plasma-containing environments in greater detail. Here, an emphasis is placed on conditions that lead to the surface decarburization of steel, including brief explanations of the possible mechanisms, means for prevention of this effect, and dominant microstructural changes occurring during this process, with an accent on the presence of ε and γ’ phases. In the fourth section, the present review study demonstrates previous research findings on how plasma nitriding affects the hardness, tribological, and corrosion properties of AISI 4140 and similar steel grades. Simultaneously, where applicable, it correlates the observed changes in the mentioned properties with the presence of ε and γ’ phases and surface decarburization phenomenon. Finally, in the fifth section, the present review study highlights important research gaps and sets general directions for the future investigations.

The present article demonstrates that the knowledge and experience related to conditions at which surface decarburization occurs in plasma-free environments (presence of hydrogen, oxygen, moisture, carbon dioxide, etc.) is by far greater compared to those in plasma-containing environments. Moreover, strategies for the prevention of surface decarburization are far less complex. In the case of plasma-containing environments, it becomes challenging to prevent surface decarburization and simultaneously achieve a favorable plasma-nitriding effect. Besides the typical quenched and tempered state, future studies should include the surface-decarburized state as a reference state when evaluating the effects of plasma nitriding on the properties of AISI 4140 and similar steel grades. The crystallographic and other microstructural effects should be more extensively investigated. Emphasis should be placed on examining the surface decarburization effect from a material-oriented perspective rather than the process-focused approach commonly adopted to date. The studies should also consider new approaches for increasing the content and stability of ε nitrides due to their positive effects on hardness, tribological, and corrosion properties of AISI 4140 and similar steel grades. Efforts should be made towards quantifying and distinguishing the effects of surface decarburization achieved before and during plasma nitriding on the final properties of plasma-nitrided steels.

## Figures and Tables

**Table 1:** Chemical composition of 42CrMo steel. Reprinted from [2], with permission from Elsevier.

Element	C	Si	Cr	Mn	Mo	P	S	Fe
wt.%	0.38–0.45	0.20–0.40	0.90–1.20	0.50–0.80	0.15–0.25	≤0.04	≤0.04	Bal.

**Table 2:** Summary of plasma-nitriding parameters and results of Cesconetto et al. [130].

**1. Material**	**2. State of the Material Before Nitriding**	**3. Nitriding Condition**	**4. Chemico-Structural** **Changes**
API 5L X-70	Pearlite in a matrix of ferrite was wet-grounded using SiC papers and mechanically polished to a mirror-like finish	Plasma cleaning: temperature of 200–250 °C, Ar atmosphere, pressure of 100 Pa, duration of 15 min. Plasma nitriding: temperatures of 410, 440 and 470 °C, nitriding times of 1, 3 and 5 h, pressure of 533 Pa, gas composition of 10% N_2_ and 90% H_2_	During 1 h nitriding of the sample, both ε-Fe_2-3_N and γ’-Fe_4_N nitrides were detected, and during 3 and 5 h nitriding of the samples only γ’-Fe_4_N nitride was evidenced
**5. Methods for monitoring chemical–structural changes**	**6. Tribology**	**7. The most favorable structure from the aspect of tribology**	
SEM, XRD, “free ball” micro-abrasion tester, micro-HV	Ball-on-disc tribometer was used. Counter-body was an AISI 52100 steel ball with 25.4 mm in diameter. Abrasive slurry was 4.5 μm SiC particles. Normal load was 0.24 N. Test samples rotated at 150 rpm	The maximum wear resistance was achieved when the compound layer consisted mainly of ε-Fe_2-3_N nitride and a diffusion zone with large needle-like γ’-Fe_4_N nitride at 440 °C for 1 h	

**Table 3:** Summary of plasma-nitriding parameters and results of Zhang et al. [135].

**1. Material**	**2. State of the Material Before Nitriding**	**3. Oxynitro-Carburization Condition**	**4. Chemico-Structural Changes**	**5. Methods for Monitoring Chemico-Structural Changes**
35CrMo	Austenitized at 860 °C for 1800 s, quenched in oil, tempered at 580 °C for 1800 s, and air-cooled	Oxynitro-carburization was carried out at 550, 570 and 610 °C for 2 h, with a gas mixture of NH_3_, O_2_, and additive organic gas at 0.11 MPa in a low-temperature gas multi-element penetrating system. The samples were cooled in air	Microstructure, surface composition, case depth, microhardness, wear, and corrosion resistance of the γ’-Fe_4_N, ε-Fe_3_N, Fe_3_O_4_, and Fe_2_O_3_	XRD, wear testing, corrosion test, and microhardness
**6. Corrosion**	**7. Tribology**	**8. The most favorable structure from the aspect of corrosion**	**9. The most favorable structure from the aspect of tribology**	
Salt spray test system was used to evaluate the corrosion resistant behavior after surface treatment	Block-on-ring tribometer was employed. Counter-body was a GCr15 steel. Normal load was 200 N, sliding speed was 0.4 m/s, and a sliding distance was 251 m	The properties of samples treated at 570 °C were proved to be the best	The best wear resistance property is obtained from the 570 °C treated sample. Resistance increases with increasing ε-Fe_3_N	

## Data Availability

The original contributions presented in this study are included in the article. Further inquiries can be directed to the corresponding authors.
